# Composition and functionalities of peanut protein concentrates using ethanol as a defatted solvent and a precipitating agent

**DOI:** 10.1002/jsfa.70967

**Published:** 2026-08-07

**Authors:** Paloma Jamily Cristina Magalhães, Luiza Cardoso Stumm, Paola de Cássia Franco Visioli, Keila Kazue Aracava, Rafaela Torrezan Privatti, Maria Carolina Capellini, Silvana Marina Piccoli Pugine, Daniel Gonçalves, Christianne Elisabete da Costa Rodrigues

**Affiliations:** ^1^ Laboratório de Engenharia de Separações (LES), Departamento de Engenharia de Alimentos (ZEA), Faculdade de Zootecnia e Engenharia de Alimentos (FZEA) Universidade de São Paulo (USP) São Paulo Brazil; ^2^ Laboratório de Química Biológica (LQB), Departamento de Ciências Básicas (ZAB), Faculdade de Zootecnia e Engenharia de Alimentos (FZEA) Universidade de São Paulo (USP) São Paulo Brazil; ^3^ Laboratório de Tecnologia de Alimentos (LTA), Centro de Ciências e Tecnologias Agropecuárias (CCTA) Universidade Estadual do Norte Fluminense Darcy Ribeiro (UENF) Rio de Janeiro Brazil

**Keywords:** *Arachis hypogaea* L., defatted meal, protein precipitation, protein purification, protein structure, techno‐functional properties

## Abstract

**BACKGROUND:**

Defatted solids (DSs) from vegetable oil extraction represent valuable feedstocks for producing protein concentrates with tailored functional properties. In the present study, using peanut protein concentrates obtained from press cakes defatted with ethanol (DSEt) or hexane (DSHx), we aimed to evaluate ethanol as a green solvent for oil extraction and as a protein‐precipitating agent. Protein extraction was optimized by assessing temperature and pH, and purification was performed using isoelectric (IP) or alcoholic (AP) precipitation.

**RESULTS:**

Extractions at 75 °C and a pH of 9.0 resulted in higher protein extraction yields for DSEt (89 ± 3%) and DSHx (95 ± 1%). The protein contents reached 780 ± 30 g kg^−1^ (IP) and 790 ± 10 g kg^−1^ (AP) for DSEt and 730 ± 30 g kg^−1^ (IP) and 750 ± 10 g kg^−1^ (AP) for DSHx (dry basis). Both the solvents and the precipitation methods maintained the thermal stability of the proteins, although the denaturation enthalpies indicated that IP was less severe than AP. Compared with DSHx‐IP (88 ± 4% and 43.0 ± 0.1%, respectively), the DSEt‐AP proteins showed higher solubility (99.84 ± 0.01%), foam formation (71.0 ± 0.1%) and comparable emulsifying and water‐ and oil‐absorption capacities. Fourier transform infrared spectroscopy indicated that the ethanol used in defatting altered the hydrophilic/hydrophobic balance, exposing more hydrophilic groups regardless of the precipitation method. AP reduced the protein hydrophobicity more strongly in DSHx solids, possibly because of depolymerization/aggregation of arachin subunits.

**CONCLUSION:**

The use of ethanol in both the defatting and precipitation stages enables the production of protein concentrates with functional properties equivalent to those of conventional hexane‐IP methods at the same time as using a greener, safer solvent. © 2026 The Author(s). *Journal of the Science of Food and Agriculture* published by John Wiley & Sons Ltd on behalf of Society of Chemical Industry.

## INTRODUCTION

The demand for sustainable and protein‐rich foods drives innovation in the food industry. Oilseeds are key sources of lipids and proteins, and their processing yields coproducts that can be transformed into affordable nutrient sources.^1^, ^2^, ^3^

Peanuts (*Arachis hypogaea* L.) are the fourth most cultivated oilseeds globally, with production reaching 52.23 million metric tons in February 2026, mainly in China and India.^3^ The high lipid content (400–500 g kg^−1^) of peanuts allows oil to be obtained by mechanical pressing, yielding a press cake. Residual oil can be further extracted from the press cake using solvents, whereas the remaining solid fraction constitutes the peanut meal or defatted peanut solid.^1^, ^4^

The industrial extraction of residual oil from peanut press cake uses a mixture of *n*‐hexane isomers, known as ‘hexane’, which is obtained from the distillation of naphtha, a volatile petroleum fraction. Despite its high solubilization capacity for vegetable oils and easy recovery, hexane has several disadvantages. As a result of its high volatility, losses of hexane during the process can contribute to environmental pollution, and hexane is also highly flammable and toxic and is categorized as Class 2 (meaning its use should be limited to 2.9 mg day^−1^ because of its high toxicity and neurotoxic effect).^5^ Studies have suggested the replacement of hexane by alternative solvents in the vegetable oil extraction step, with the goal of mitigating the adverse effects of hexane. This replacement is linked to the UN Sustainable Development Goals SDG 12 (Responsible Consumption and Production) and SDG 13 (Climate Action). Ethanol stands out from other compounds, as shown in the compilations reported by Carré *et al*.^6^

Ethanol can be obtained from renewable sources (bioethanol), being toxic than hexane, and is classified as Class 3 (low risk to human health, allowing residual amounts of 50 mg day^−1^).^5^ The USA and Brazil were responsible for approximately 80% of the 31.2 million gallons of ethanol produced worldwide in 2024.^7^

The use of ethanol has been evaluated for oil extraction from soybean (*Glycine max*), rapeseed (*Brassica napus*), and press cakes of baru (*Dipteryx alata*), macadamia (*Macadamia integrifolia*) and peanut (*Arachis hypogaea* L.).^8^, ^9^, ^10^, ^11^, ^12^ In these studies, the impact of ethanol on the protein fraction contained in the defatted solid obtained after solvent extraction was also evaluated in terms of the nitrogen solubility index.

Peanut meal or defatted peanut solid contains approximately 500 g kg^−1^ protein and is rich in essential amino acids such as arginine. In February 2026, their production reached 7.78 million metric tons compared to 288.94 million metric tons of soybean meal.^3^ Despite their nutritional value, these solids are mostly used as feed because of challenges in protein extraction.^2^ Nonetheless, they represent a promising raw material for producing protein concentrates or isolates for food fortification, adding value to the peanut chain.^13^

Peanut proteins have been extracted from peanut meal defatted with solvents such as *n*‐hexane^14^ and pressurized butane or propane.^15^ However, studies using defatted solids with ethanol for protein extraction remain scarce or non‐existent. Despite the potential of ethanol to replace hazardous, fossil‐derived solvents, its use is challenging because it can denature proteins during oil extraction.^10^, ^11^, ^12^

Peanut protein concentrates can be obtained via the pH‐shift method, which involves extraction with water under alkaline conditions, followed by isoelectric precipitation of the solubilized proteins.^14^, ^16^ Wu *et al*.^15^ evaluated peanut protein extraction from solids defatted with butane and propane. Different protein extraction methods were tested using water and alkaline solutions as solvents, followed by isoelectric precipitation to recover the proteins. Wu *et al*.^15^ also washed with ethanol to increase protein purity.^15^ Although isoelectric precipitation is fast, easy to perform and enables high protein yields, it becomes necessary to use large volumes of acids and alkalis, generating toxic waste. Therefore, alternative methods are desirable when agents that promote molecular aggregation, water miscibility and non‐toxicity are available.^17^ Ethanol has been proposed as a low‐cost, recoverable precipitating agent and has been successfully applied to purify inulinase, bromelain, pectin and proteins from potato and okara.^18^, ^19^, ^20^, ^21^, ^22^ Alcoholic precipitation exploits the ability of ethanol to disrupt hydrophobic protein interactions, reducing solubility in water.^19^, ^20^, ^21^, ^22^

Thus, there is a need to investigate whether the use of ethanol to defeat the oleaginous matrix and to precipitate proteins extracted from defatted solids can yield a protein concentrate with high protein content and improved functional properties. Evaluation of the effects of ethanol on techno‐functional properties, such as solubility, water‐ and oil‐holding capacity, emulsifying ability, and foam stability, is crucial for potential industrial applications.^15^

In the present study, ethanol was assessed as a protein‐precipitating agent, and its effects on the composition and functional properties of peanut protein concentrates were compared with those obtained by conventional isoelectric precipitation using solids defatted with either ethanol or *n*‐hexane. The dual use of ethanol for defatting and protein precipitation represents an innovative and sustainable approach within the peanut value chain.

## MATERIALS AND METHODS

### Materials

Peanut press cake (PPC) (Sr. Ouro Verde, Almirante Tamandaré, Paraná, Brazil) was defatted via a sequential cross‐current extraction process using absolute ethanol or hexane, according to the method of Magalhães *et al*.^12^ For hexane solvent, extraction was performed in two stages at 55 °C, with a PPC‐to‐hexane mass ratio of 1:4, yielding a peanut oil extraction yield of 87 ± 5%. With respect to ethanol, oil extraction was performed in three stages at 75 °C, with a PPC‐to‐ethanol mass ratio of 1:5, yielding an oil extraction yield of 81 ± 4%. The resulting defatted solids were designated DSEt (ethanol) and DSHx (hexane).

The reagents included sodium hydroxide, absolute ethanol, *n*‐hexane, glacial acetic acid, anhydrous sodium carbonate, refined soybean oil, hydrochloric acid, gallic acid, Folin–Ciocalteu solution, bromophenol blue, acrylamide, *bis*‐acrylamide, *tris*‐acrylamide, ammonium persulfate, tetramethylethylenediamine, sodium dodecyl sulfate (SDS), glycerol, *β*‐mercaptoethanol, glycine, Coomassie Brilliant Blue R‐250, multicolor protein standards (10–260 kDa) and acetone. The purities of all the reagents were greater than 98%.

### Experimental procedure

The experimental setup for protein extraction and precipitation conditions is schematically illustrated in the flowchart of Fig. 1. A schematic design of the trials highlighting the use of ethanol for oil extraction and protein precipitation is shown in the Supporting information (Fig. S1).

**Figure 1:**
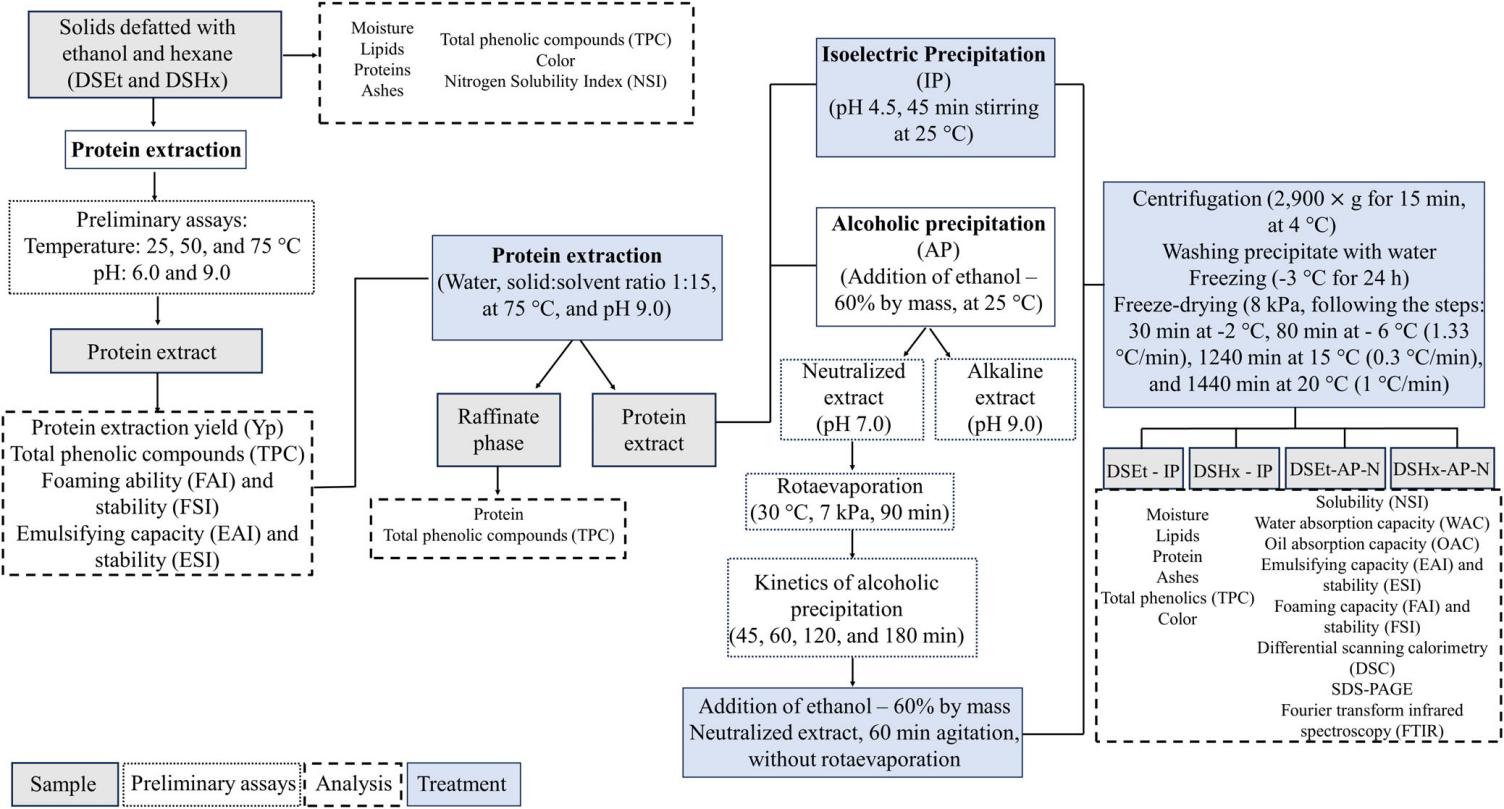
Experimental setup for protein extraction and precipitation.

#### Characterization of defatted solids

DSEt and DSHx were obtained via sequential extractions as described by Magalhães *et al*.^12^ DSEt was obtained with three ethanol extractions at 75 °C (solvent/PPC mass ratio of 5:1) and DSHx was obtained with two hexane extractions at 55 °C (4:1 mass ratio).

DS characterization also followed Magalhães *et al*.^12^ The moisture content was determined by heating in a convection oven at 130 °C for 4 h (Ac 2–41^23^). Lipids were assessed by the rapid method for total lipid determination in a semi‐automatic pressurized extractor (XT10’; Ankom Technology, Macedon, NY, USA), using hexane and heating at 90 °C for 1 h (Am 5‐04^23^). Total nitrogen was quantified by combustion (FP‐528; Leco, St Jospeh, MI, USA) (Ba 4e‐93^23^) and then converted to peanut protein using a factor 5.46, as recommended by the AOCS for peanuts.^23^ The ash content was measured by incineration (Ba 5a‐49^23^) and carbohydrate content was estimated by difference on a dry basis.

The total phenolic content (TPC) in DSEt and DSHx was assessed following the methods of Ortega *et al*.^24^ using a gallic acid calibration curve (mg GAE g^−1^). Phenols were extracted with acetone/water/glacial acetic acid (70/29.5/0.5, v/v/v) from 0.5 g of cold‐defatted sample (solid/hexane ratio 1:3) by performing three stirring (MR Hei‐Tec; Heidolph, Schwabach, Germany) and resting cycles (5 and 25 min) with ultrasonication (UltraCleaner 1400; Unique, São Paulo, Brazil) at 37 °C for 10 min. Extracts were centrifuged (CR3i; Thermo Jouan, Saint‐Herblain, France) (3000 × *g* for 5 min at 25 °C) and filtered (0.45‐μm Alcrom nylon syringe filters). The reaction mixture contained 0.5 mL of extract, 2.5 mL of 100 g kg^−1^ Folin–Ciocalteu reagent and 2.0 mL of 75 g kg^−1^ Na₂CO₃. After 2 h in the dark, the absorbance was read at 760 nm (UV‐1650 PC; Shimadzu, Kyoto, Japan).

The protein solubility of DSEt and DSHx was assessed as the nitrogen solubility index (NSI, %) by following the method of Morr *et al*.^25^ and using deionized water (25 °C, pH 9.0):

(1) $$\mathrm{NSI}\;\left(\%\right)=\frac{N_{\text{liquid phase}}\times m_{\text{water}}}{N_{\text{sample}}\times m_{\text{sample}}}\times 100$$

where *N*_*liquid phase*_ and *N*_*sample*_ are the nitrogen contents (g kg^−1^) in the filtrate and solid, respectively, and *m*_*water*_ and *m*_*sample*_ are the water and sample masses (g), respectively.

The instrumental color of the defatted solids (DSs) was determined using a non‐contact reflectance spectrophotometer (Aeros; HunterLab, Reston, VA, USA) through L* (lightness), a* (red/green) and b* (yellow/blue) coordinates. The hue angle (h°) was calculated as:

(2) $$h^{{}^{\circ}}=\tan^{-1}\frac{b^{*}}{a^{*}}$$

The water absorption capacity (WAC) and oil absorption capacity (OAC) of PPC, DSEt, and DSHx were determined by following the methods of Wu *et al*.^15^ with minor modifications. Briefly, 0.5 g of sample was mixed with 20 g of deionized water or oil, shaken for 60 min, and centrifuged at 3000 × *g* for 30 min (25 °C). The supernatant was discarded, and the WAC and OAC were expressed as grams of water and oil per gram of protein, respectively.

#### Preliminary assays for protein extraction

Preliminary protein extraction assays were conducted using DSs to evaluate the effects of temperature (25, 50 and 75 °C) and pH (6.0 and 9.0). A pH of 6.0 corresponds to the natural DS value, whereas a pH of 9.0 increases peanut protein solubility.^15^ The protein content and TPC of the solid residues was characterized as described in the section on the Characterization of defatted solids. The protein content, TPC, emulsification capacity and foaming capacity of the extracts obtained under each condition were analyzed (see section on the Characterization of the extracts from the preliminary protein extraction assays).

##### Characterization of the extracts from the preliminary protein extraction assays

The protein extracts obtained at 25, 50 and 75 °C and a pH of 6.0 or 9.0 were vacuum filtered under 21 kPa, and the solubilized proteins were quantified by total nitrogen (Ba 4e‐93^23^) as described in the section on the Characterization of defatted solids. The protein extraction relative yield (Yp, %), defined as the protein yield in proportion to the protein content in the defatted solid, was calculated according to:

(3) $$Y_{p}\;\left(\%\right)=\frac{N_{\text{extract}}\times m_{\text{extract}}}{N_{\mathrm{DS}}\times m_{\mathrm{DS}}}\times 100$$

where *N*_*extract*_ and *N*_*DS*_ are the nitrogen contents of the protein extract and defatted solid (g kg^−1^), respectively, and *m*_*extract*_ and *m*_*DS*_ are the masses of solvent and defatted solid (kg), respectively.

The TPC of the protein extracts was determined as described in the section on the Characterization of defatted solids, and the extract dilution process was adjusted. The samples were diluted in deionized water at 1:125 (w/w) for the pH 6.0 extracts and at 1:250 (w/w) for the pH 9.0 extracts.

The emulsifying activity (EAI) and stability (ESI) were measured following the method of Pearce & Kinsella.^26^ The extracts were adjusted to 1 g kg^−1^ protein in deionized water, mixed with 1 mL of soybean oil and homogenized (Ultraturrax; Ika, Staufen, Germany; 10 000 rpm for 1 min). An aliquot (200 μL) was added to 12.5 mL of 1 g kg^−1^ SDS, and the absorbance was read at 500 nm. For ESI, the absorbance was remeasured after 10 min. The EAI and ESI were calculated as shown in Eqns (4) and (5), respectively:

(4) $$\mathrm{EAI}\;\left(m^{2}/g\right)=\frac{2\times 2,303\times A_{t}\times D}{C\times \emptyset \times 10000}$$

(5) $$\mathrm{ESI}\;\left(\%\right)=\frac{E{\mathrm{AI}}_{t=10}}{{\mathrm{EAI}}_{t=0}}\times 100$$

where *A*_*t = 0*_ and *A*_*t = 10*_ are the absorbances immediately after emulsion formation (*t* = 0) and after 10 min (*t* = 10), respectively; *D* is the dilution factor (62.5); *C* is the protein concentration (kg m^−3^); and *Ø* is the oil volume fraction in the emulsion.

The foaming activity index (FAI) [Eqn (6)] was calculated by following the method of Aracava *et al*.^10^ The extracts were adjusted to 10 g kg^−1^ protein and homogenized (Ultraturrax; 10 000 rpm for 5 min). Foam height was measured before (*A*₁) and after (*A*₂) formation for calculation of FAI:

(6) $$\mathrm{FAI}\;\left(\%\right)=\frac{A_{2}-A_{1}}{A_{1}}\times 100$$

The foaming stability index (FSI) was determined by measuring the foam height [foam + suspension (cm)] at 10, 30, 45 and 60 min.

#### Protein extraction experiments

The extraction conditions were based on preliminary assays (see section on Preliminary assays for protein extraction). Peanut proteins were extracted in jacketed borosilicate glass cells at 75.0 ± 0.1 °C using DSEt or DSHx dispersed in deionized water (1:15, kg kg^−1^) with constant stirring (400 rpm, magnetic stirrer). The pH was adjusted to 9.0 with 2 n NaOH. After 100 min, the mixture was centrifuged (2900 × *g* for 15 min at 4 °C), yielding a liquid protein extract and solid raffinate. The raffinates were dried at 100 °C for 24 h, after which the TPC, lipid and protein contents were determined (see section on Characterization of defatted solids). The protein extracts were characterized as described in the sections on Characterization of the extracts from the preliminary protein extraction assays and Protein precipitation, respectively

#### Protein precipitation

##### Isoelectric precipitation

Isoelectric precipitation (IP) was performed by followed the methods of Wu *et al*.^15^ with modifications. The protein extracts were vacuum‐filtered, adjusted to pH 4.5 with 2 n HCl, and stirred (400 rpm at 25 °C) for 45 min. The mixture was centrifuged (2900 × *g* for 15 min at 4 °C), the supernatant was discarded, and the precipitate was washed three times with deionized water and centrifuged (2900 × *g* for 10 min at 4 °C). The precipitate was redispersed, neutralized with 2 n NaOH, frozen at −3 °C for 24 h, and lyophilized (single‐chamber equipment, Scientz‐10ND; Ningbo Scientz Biotechnology Co. Ltd., Ningbo, China) through sequential temperature steps as described by Privatti *et al*.^27^ Protein concentrates from DSEt and DSHx were designated DSEt‐IP and DSHx‐IP, respectively.

##### Alcoholic precipitation

For alcoholic precipitation (AP), absolute ethanol was added to protein extracts at a ethanol to protein extract mass ratio of 60:40, yielding 600 g⋅of ethanol per kg^−1^ of solution.^22^ The solution was stirred (400 rpm at 25 °C) for 45 min, and then centrifuged, washed, frozen and lyophilized as described in the section on Isoelectric precipitation.

1. *Effect of extract neutralization before and after alcoholic precipitation*: Precipitation was evaluated with extracts neutralized before ethanol addition (denoted DSEt‐AP‐N or DSHx‐AP‐N) and with alkaline extracts without pH adjustment (DSEt‐AP‐A or DSHx‐AP‐A).
2. *Effect of the rotary evaporation of ethanol on the protein content*: After neutralization and ethanol addition, the extracts were subjected to rotary evaporation (Hei‐VAP Silver; Heidolph; 30 °C; 7 kPa; ~90 min) to reduce dilution and increase the protein concentration. The subsequent steps were as above, yielding DSEt‐AP‐N‐R or DSHx‐AP‐N‐R.
3. *Alcoholic precipitation kinetics*: Agitation times of 45, 60, 120 and 180 min were tested for neutralized extracts (AP‐N) to determine the minimum time for maximal protein precipitation, with centrifugation, washing, freezing and lyophilization subsequently performed.

#### Characterization of protein concentrates

##### Centesimal composition and color parameters

The moisture of the lyophilized protein concentrates obtained by isoelectric precipitation (DSEt‐IP and DSHx‐IP) and alcoholic precipitation with neutralized extract (DSEt‐AP‐N, DSHx‐AP‐N) was measured in a high‐temperature dryer (105 °C for 3 h; Ankom Technology, Macedon, NY, USA). The TPC, lipid content, protein content, ash content and NSI were assessed as described in the section on Characterization of defatted solids. Instrumental color was measured as described in the section on Characterization of defatted solids using DSEt and DSHx as references ($L_{0}^{*}$, $a_{0}^{*}$ and $b_{0}^{*}$) to calculate total color change (Δ*E*):

(7) $$∆E=\sqrt{{\left(L^{*}-L_{0}^{*}\right)}^{2}+{\left(a^{*}-a_{0}^{*}\right)}^{2}+{\left(b^{*}-b_{0}^{*}\right)}^{2}}$$

##### Functional properties

The functional properties of the protein concentrates were analyzed in terms of the EAI, ESI, FAI and FSI, as described in the section on Characterization of the extracts from the preliminary protein extraction assays. The WAC and OAC were evaluated as detailed in the section on Characterization of defatted solids.

##### Differential scanning calorimetry (DSC)

The thermal behavior of the proteins was analyzed by DSC (TA2010; TA Instruments, New Castle, DE, USA) by following the method of Navarro & Rodrigues.^11^ Lyophilized proteins were prepared as 500 g kg^−1^ slurries in deionized water and equilibrated at 25 ± 2 °C for 24 h. The samples were sealed in aluminum pans and heated from 20 to 120 °C (10 °C min^−1^) in the DSC instrument, with an empty pan used as reference and indium used for calibration. The denaturation enthalpy (Δ*H*, J g^−1^ protein) and denaturation temperature (*T*_d_, °C) were calculated using Universal Analyze V.3.9A (TA Instruments). The DSC curve of PPC was also measured for comparison.

##### Sodium dodecyl sulfate‐polyacrylamide gel electrophoresis (SDS‐PAGE)

The molecular profile of the peanut protein concentrates was analyzed by SDS‐PAGE.^22^ Neutralized protein solutions (2 kg m^−3^) were mixed 1:1 (w/w) with buffer (Tris‐HCl, SDS, glycerol, *β*‐mercaptoethanol, bromophenol blue) and heated at 95 °C for 5 min, after which 25‐μL aliquots were loaded onto 125 g kg^−1^ separation gels per 50 g kg^−1^ stacking gels. Electrophoresis was performed at 110 V for 240 min with a 10–260‐kDa multicolor standard. The gels were stained with 1 g kg^−1^ Coomassie blue, destained with methanol/acetic acid (59/41, v/v), and imaged using ChemiDoc MP (Bio‐Rad, Hercules, CA, USA); the images were converted to black and white for clarity.

##### Fourier transform infrared spectroscopy (FTIR)

The secondary structure of the proteins in PPC, DSEt, DSHx and protein concentrates was analyzed by FTIR (Cary 630; Agilent, Santa Clara, CA, USA) using ATR with a ZnSe crystal (MicromATR; Czitek, Danbury, CT, USA) and MicroLab PC software (Agilent). Spectra were recorded from 650–4000 cm^−1^ at a resolution of 16 cm^−1^ (64 scans per sample) and analyzed in Origin 2024 (OriginLab Corp., Northampton, MA, USA) by following the methods of Capellini *et al*.^28^

#### Statistical analysis

Protein extraction and precipitation trials were performed in duplicate, and characterization analyses were performed in triplicate. The results were analyzed with 95% significance using Duncan's test for multiple comparisons with the SAS software, version 9.3 (SAS Institute Inc., Cary, NC, USA) and a *t*‐test for mean comparisons with R, version 4.2.1 (Core Team, The R Foundation, Vienna, Austria).

## RESULTS AND DISCUSSION

### Characterization of defatted solids

The DSs obtained via sequential oil extraction^12^ were characterized to determine their proximate composition, NSI) and water and oil absorption capacities (WAC and OAC, respectively) (Table 1).

**Table 1:** Composition and functionalities of peanut press cake defatted with ethanol (DSEt) or hexane (DSHx)

		DSEt	DSHx
Composition (g kg^−1^)	Moisture	49 ± 1 A	28 ± 1 B
	Lipids^a^	145 ± 2 A	99 ± 6 B
	Proteins^a^, ^b^	452 ± 3 B	475 ± 4 A
	Ashes^a^	35 ± 1 A	37 ± 2 A
	Carbohydrates^a^, ^c^	368 ± 6 A	390 ± 10 A
	TPC (mg GAE g^−1^ DS)^d^	5.8 ± 0.3 B	9 ± 1 A
Functionalities	NSI (%)	86 ± 6 B	98 ± 1 A
	WAC (g water⋅g^−1^ protein)	11.7 ± 0.1 A	8.04 ± 0.01 B
	OAC (g oil⋅g^−1^ protein)	5.26 ± 0.03 A	4.4 ± 0.1 B
Color characteristics	L*	83.5 ± 0.4 A	85.8 ± 0.1 A
	a*	3.0 ± 0.1 A	1.8 ± 0.1 B
	b*	11.04 ± 0.04 A	11.5 ± 0.2 A
	*°h*	74.9 ± 0.5 B	81.0 ± 0.1 A

^a^ g kg^−1^ on a dry basis.

^b^ N × 5.46.

^c^ Calculated by difference.

^d^ Milligram of gallic acid equivalent per gram of sample.

DSEt, solid defatted with ethanol; DSHx, solid defatted with hexane; TPC, total phenolic compounds; NSI, nitrogen solubility index; WAC, water absorption capacity; OAC, oil absorption capacity. The same uppercase letters in the same row do not differ from each other (*P* > 0.05). Comparisons were carried out at the 95% confidence level using a *t*‐test.

Compared with DSEt, DSHx had a lower lipid content, most likely because of hexane's greater affinity for and solubility in triacylglycerols. Consequently, DSHx resulted in a higher protein content as a compensatory effect of defatting, whereas the ash and carbohydrate contents did not significantly differ (*P* > 0.05).

Compared with DSHx, DSEt resulted in a lower NSI. Ethanol combined with heat can denature proteins by altering their secondary, tertiary or quaternary structures and expose groups that are hidden in the native conformation of the protein. This denaturation weakens the hydrophobic interactions among non‐polar residues, thereby reducing protein solubility.^11^, ^29^ The WAC and moisture corroborate these results, as compared with DSHx and PPC (3.6 ± 0.1 g water g^−1^ protein), DSEt resulted in a higher WAC, which was likely a result of ethanol‐induced conformational changes exposing more hydrophilic groups (Table 1).

As shown in Table 1, both DSs had similarly high carbohydrate contents (*P* > 0.05), which influences the WAC because of the polar side chains with high swelling capacity.^29^ This findings supports the hypothesis that ethanol exposed protein hydrophilic groups because the carbohydrate levels were unchanged (*P* > 0.05).

DSEt and DSHx exhibited OACs that were greater than the PPC (3.2 ± 0.1 g oil g^−1^ protein), suggesting that defatting, particularly with ethanol, altered the hydrophilic–hydrophobic balance at the protein surface.

DSEt had a lower TPC (Table 1), most likely because the polarity of ethanol favors coextraction of phenolic compounds during oil extraction.^30^ Additionally, the higher extraction temperature used for oil extraction using ethanol (75 °C *vs*. 55 °C for hexane) may have enhanced compound mobility and partial degradation and thus reduced the TPC.

DSEt and DSHx differed slightly in terms of red/green (a*) values (*P* ≤ 0.05) but did not significantly differ in terms of luminosity (L*) or yellow/blue (b*) coordinates.

### Preliminary protein extraction assays

Preliminary peanut protein extraction trials (see section on Preliminary assays for protein extraction) evaluated temperature (25, 50 and 75 °C) and pH (6.0 and 9.0). The extraction yields and functional properties are shown in Fig. 2, and the protein and TPC compositions of the solid phases (raffinates) are reported in the Supporting information (Table S1).

**Figure 2:**
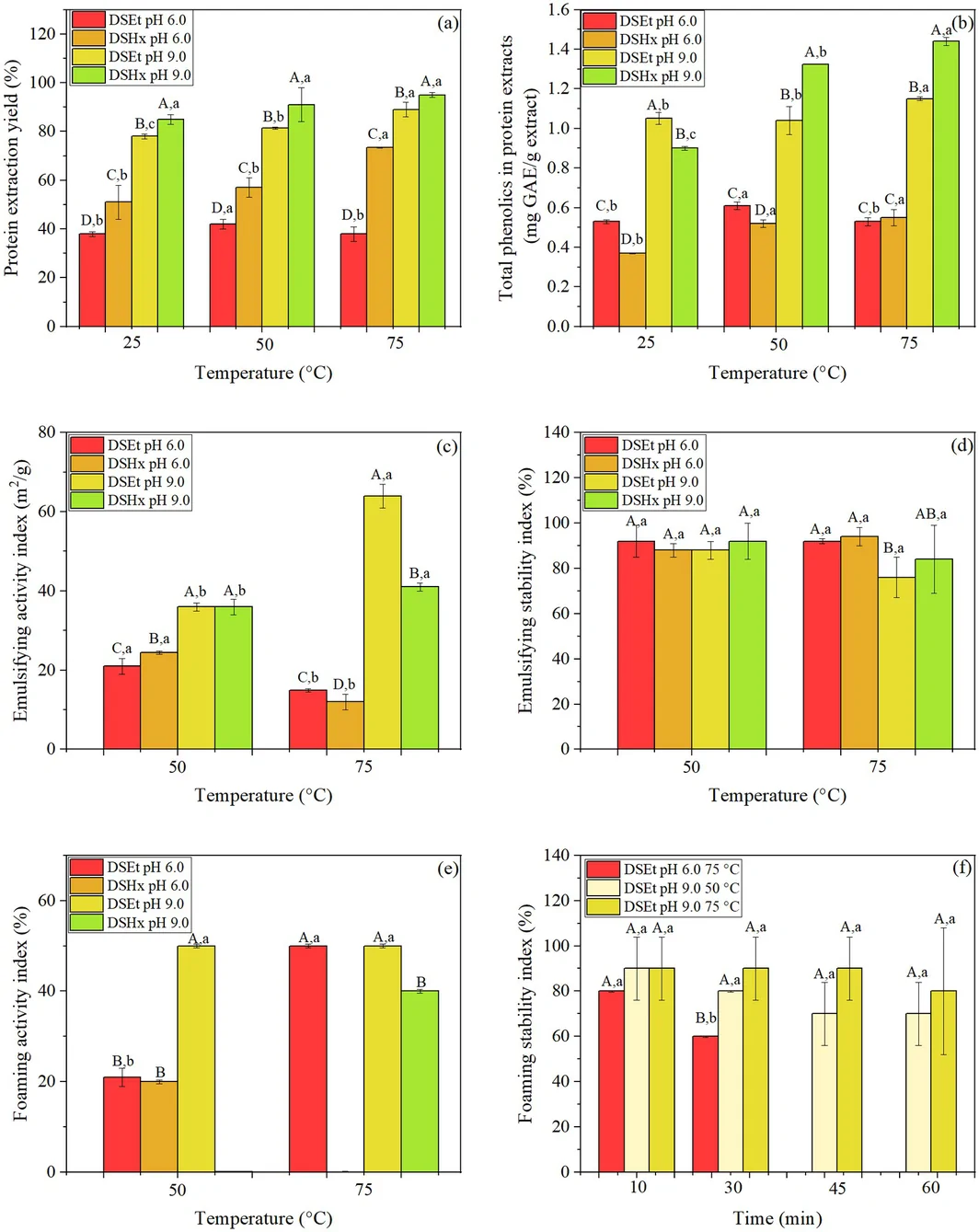
Protein extraction results from solids defatted with ethanol (DSEt) or hexane (DSHx) as a function of temperature and pH. (a) Protein extraction yield (Y_P_, %), (b) total phenolic content in protein extracts (TPC, mg GAE g^−1^ extract), (c) emulsifying activity index (EAI, m^2^ g^−1^), (d) emulsifying stability index (ESI, %), (e) foaming activity index (FAI, %) and (f) foaming stability index (FSI, %). The same uppercase letters for different pH values at the same temperature and the same lowercase letters for different temperatures at the same pH indicate no significant difference according to Duncan's test (*P* > 0.05).

High temperatures (75 °C) and alkaline pH (9.0) promoted protein extraction from both DSEt and DSHx (Fig. 2(a)), yielding extracts with a high TPC (Fig. 2(b)). Raffinates from pH 9.0 extractions presented low protein content and TPC (see Supporting information, Table S1), which is consistent with the extract results. Alkaline conditions may disrupt disulfide bonds, increase the protein solubility and promote the release of phenols bound to cell wall components.^1^ Protein extracts obtained at alkaline pH exhibited a reddish color (see Supporting information, Fig. S2), which is likely associated with the hydroxylation of aromatic rings in phenolic compounds and also explains their high TPC values.^31^

The relative protein extraction yields obtained at 75 °C and pH 9.0 (Fig. 2(a)) were consistent with the NSI values obtained for DSEt (86 ± 6%) and DSHx (98 ± 1%) (Table 1). On the basis of extraction yields (Fig. 2(a)), protein extracts obtained from DSEt and DSHx at 50 and 75 °C were further evaluated for their emulsifying and foaming properties, reflecting protein adsorption at water–oil and water–air interfaces, respectively.

#### Characterization of the extracts from the preliminary protein extraction assays

An increase in pH increased the EAI (Fig. 2(c)), most likely as a result of enhanced protein solubility, with the extracts obtained at 75 °C and a pH of 9.0 showing the highest values. Despite DSHx extracts having relatively high protein content, their EAI was lower than that of DSEt under the same conditions, which is consistent with the WAC results (see section on Characterization of defatted solids), suggesting that defatting alters the protein hydrophilic/hydrophobic balance. Ethanol and hexane may modify this balance differently, and affect protein film formation around oil droplets and emulsion stability.^4^, ^15^ Alkaline pH may also promote protein unfolding via intramolecular electrostatic repulsion, expose hydrophobic groups and increase the EAI.^1^ The emulsion stabilities were similar (*P* > 0.05) and all exceeded 70% (Fig. 2(d)).

No foam formed in the DSHx extracts obtained at 50 °C/pH 9.0 or 75 °C/pH 6.0 (Fig. 2(e)). Although the DSHx extracts produced foam at 50 °C/pH 6.0 and 75 °C/pH 9.0, they lacked stability (Fig. 2(f)), this was likely a result of the low protein solubility at pH 6.0 and the reduced WAC of DSHx (Table 1). Conversely, the DSEt extracts obtained at both temperatures and a pH of 9.0 presented high FAI values (Fig. 2(e)). Alkaline pH combined with elevated temperature (75 °C) increased the FSI (Fig. 2(f)), indicating the role of temperature in modifying protein conformation. Increased kinetic energy promotes molecular unfolding and reorganization at the interface, enabling the formation of a continuous protein film that decreases surface tension and supports foam stability.^29^

On the basis of the *Y*_*p*_ (Fig. 2(a)), TPC (Fig. 2(b)) and protein functionality results (Fig. 2(c)–(f)), 75 °C and a pH of 9.0 were selected as the optimal conditions for protein extraction from both DSEt and DSHx.

### Protein extraction and precipitation strategies

Peanut proteins extracted from DSEt and DSHx at 75 °C and pH 9.0 were purified by IP and AP. The protein content and color parameters of the resulting concentrates are shown in Table 2.

**Table 2:** Protein content and colorimetric analysis of protein concentrates obtained by different precipitation methods from solids defatted by ethanol (DSEt) or hexane (DSHx)

Raw material	Treatment		Protein content (g kg^−1^, dry basis)	Color parameters				
				L*	a*	b*	Δ*E*	*h*°
DSEt	IP		780 ± 30 A,a	32.1 ± 0.4 B,b	14 ± 2 A,a	10 ± 2 A,b	52.6 ± 0.4 A,b	36 ± 2 A,b
	AP‐N		770 ± 20 A,a	29 ± 2 B,b	13 ± 1 B,a	10.2 ± 0.2 B,b	56 ± 2 A,a	37 ± 1 B,b
	AP‐A		660 ± 60 A,b	32 ± 1 A,b	14 ± 2 A,a	12 ± 2 A,ab	53 ± 1 B,b	41 ± 1 A,a
	AP‐N‐R		780 ± 20 A,a	37 ± 2 B,a	16 ± 1 A,a	14 ± 2 A,a	49 ± 2 B,c	40 ± 2 B,a
DSHx	IP		730 ± 30 B,b	34.7 ± 0.1 A,b	13.82 ± 0.01 A,b	11.3 ± 0.5 A,b	52.51 ± 0.02 A,b	39 ± 1 A,c
	AP N		730 ± 20 B,b	41 ± 2 A,a	16.6 ± 0.2 A,a	16.0 ± 0.4 A,a	47 ± 1 B,c	44 ± 1 A,b
	AP A		670 ± 10 A,c	26 ± 1 B,c	13.7 ± 0.4 A,b	11 ± 1 A,b	61 ± 1 A,a	38 ± 1 B,c
	AP N R		796 ± 3 A,a	44 ± 2 A,a	13.7 ± 0.4 B,b	16.2 ± 0.2 A,a	44 ± 2 A,d	49.8 ± 0.3 A,a
Alcoholic precipitation kinetics (AP‐N)	DSEt	45 min	770 ± 20 A,a	29 ± 2 B,b	13 ± 1 B,a	10.2 ± 0.2 B,b	56 ± 2 A,a	37 ± 1 B,b
		60 min	790 ± 10 A,a	27 ± 2 B,b	13 ± 2 A,ab	11 ± 2 A,a	58 ± 3 A,a	38 ± 1 B,b
		120 min	810 ± 10 A,a	32 ± 1 B,a	16 ± 1 A,a	13 ± 1 A,a	53 ± 1 A,b	39.9 ± 0.3 B,a
		180 min	784 ± 2 A,a	33 ± 1 B,a	15.4 ± 0.3 A,a	13 ± 1 A,a	52.4 ± 0.3 A,b	41 ± 2 A,a
	DSHx	45 min	730 ± 20 B,b	41 ± 2 A,a	16.6 ± 0.2 A,a	16.0 ± 0.4 A,a	47 ± 1 B,c	44 ± 1 A,b
		60 min	750 ± 10 B,a	35.2 ± 0.3 A,b	15.33 ± 0.04 A,b	12.9 ± 0.4 A,a	52.4 ± 0.4 B,a	40 ± 1 A,b
		120 min	760 ± 10 B,a	44 ± 3 A,a	16.4 ± 0.2 A,a	14.8 ± 0.4 A,a	45 ± 3 B,b	42.0 ± 0.4 A,a
		180 min	760 ± 10 B,a	40 ± 1 A,a	16 ± 1 A,a	14 ± 1 A,a	48.0 ± 0.3 B,b	40.8 ± 0.2 A,b

DSEt, solid defatted with ethanol; DSHx, solid defatted with hexane; DSEt‐IP/DSHx‐IP, protein obtained by isoelectric precipitation from solid defatted with ethanol or hexane; DSEt‐AP/DSHx‐AP, protein obtained by alcoholic precipitation from solid defatted with ethanol or hexane; DSEt‐AP‐N/DSHx‐AP‐N, protein obtained by alcoholic precipitation with neutralized extract from solid defatted with ethanol or hexane; DSEt‐AP‐A/DSHx‐AP‐A, protein obtained by alcoholic precipitation with alkaline extract from solid defatted with ethanol or hexane; DSEt‐AP‐N‐R/DSHx‐AP‐N‐R, protein obtained by alcoholic precipitation with neutralized extract and application of rotary evaporation from solid defatted with ethanol or hexane. The same uppercase letters for the same treatment and different DS, and the same lowercase letters for the same DS in different treatments indicate no statistical difference by the Duncan's yest (*P* > 0.05).

IP yielded protein concentrates of 780 ± 30 g kg^−1^ (DSEt‐IP) and 730 ± 30 g kg^−1^ (DSHx‐IP, dry basis), consistent with Gayol *et al*.,^16^ who reported 775–809 g kg^−1^ peanut protein concentrates obtained by IP under alkaline conditions.

The extract pH influenced the final protein content in AP. Neutralized extracts (AP‐N) produced higher protein contents (730–770 g kg^−1^) than alkaline extracts (AP‐A) (660–670 g kg^−1^) for both DSEt and DSHx, likely as a result of the reduced protein solubility at neutral pH. Given these higher values, DSEt‐AP‐N and DSHx‐AP‐N were subjected to rotary evaporation (R) to assess whether a reduction in ethanol dilution could promote protein precipitation.

With respect to DSEt, the protein contents did not significantly differ (*P* > 0.05) among IP, AP‐N and AP‐N‐R. In contrast, compared with IP and AP‐N, rotary evaporation increased the protein content (DSHx‐AP‐N‐R) (*P* ≤ 0.05), likely because the longer protein–ethanol contact time favored agglomerate formation.

An AP kinetic study (45–180 min) revealed that extending agitation from 45 to 60 min increased the protein content only for DSHx, with no further significant changes at 120 or 180 min.

Colorimetric analysis (Table 2) revealed that the precipitation treatments altered the color of the protein concentrates, with ΔE values ranging from 44 (DSHx‐AP‐N‐R) to 61 (DSHx‐AP‐A) (see Supporting information, Fig. S3). Ethanol precipitation reduced the luminosity (L*) and redness (a*) in some treatments (*P* ≤ 0.05). As reported by Hariharan *et al*.,^1^ ethanol can extract carbohydrates, free amino acids, and pigments, thereby affecting color. Overall, samples exhibited hue angles (*h°*) within the red‐yellow range (Table 2; see also Supporting information, Fig. S3), which is consistent with the appearance of extracts obtained at pH 9.0 (see Supporting information, Fig. S2).

On the basis of the protein content and colorimetric results, AP can be effectively performed by neutralizing the extract before ethanol addition and applying 60 min of agitation, without the rotary evaporation step. Protein concentrates for further characterization were thus prepared under these conditions (see section on Characterization of protein concentrates), along with those obtained by IP.

### Characterization of protein concentrates

#### Composition of protein concentrates

The compositions of the protein concentrates obtained by IP (DSEt‐IP and DSHx‐IP) and by AP with neutralized extracts (DSEt‐AP‐N and DSHx‐AP‐N) are shown in Table 3. All the samples can be classified as protein concentrates (730–780 g protein kg^−1^).^1^ The use of ethanol as a precipitating agent resulted in protein contents that were statistically similar to those obtained by conventional IP (*P* > 0.05). However, the TPC of the AP concentrates was lower than that of IP concentrates (*P* ≤ 0.05). Similar to the defatting step, the use of ethanol during AP likely reduced the TPC because of its high polarity and extraction capacity.^30^ Conversely, Asen *et al*.^32^ reported comparable TPC values in peanut flour defatted with acetone and in protein concentrates obtained by alkaline extraction followed by IP (0.06 mg GAE g^−1^ dry sample).

**Table 3:** Composition and functional properties of protein concentrates obtained by isoelectric precipitation (IP) and alcoholic precipitation with neutralized extract (AP‐N) from solids defatted with ethanol (DSEt) or hexane (DSHx)

			Isoelectric precipitation (IP)		Alcoholic precipitation (AP)	
			DSEt‐IP	DSHx‐IP	DSEt‐AP‐N	DSHx‐AP‐N
Composition (g kg^−1^)	Moisture		170 ± 10 ^A,a^	120 ± 10 ^B,b^	160 ± 10 ^A,a^	190 ± 20 ^A,a^
	Lipids^a^		8 ± 1 ^B,b^	12 ± 1 ^A,a^	11.0 ± 0.3 ^A,a^	11 ± 1 ^A,a^
	Proteins^a^, ^b^		780 ± 30 ^A,a^	730 ± 30 ^A,a^	750 ± 10 ^A,a^	740 ± 20 ^A,a^
	Ashes^a^		14 ± 1 ^B,b^	21 ± 1 ^B,a^	21 ± 1 ^A,b^	30 ± 2 ^A,a^
	Carbohydrates^a^, ^c^		198 ± 32 ^B,b^	237 ± 32 ^A,a^	218 ± 11 ^A,a^	220 ± 20 ^A,a^
	TPC^d^ (mg GAE⋅g^−1^ sample)		0.67 ± 0.03 ^A,b^	0.86 ± 0.04 ^A,a^	0.27 ± 0.01 ^B,a^	0.29 ± 0.02 ^B,a^
Functional properties	NSI (%)		93.54 ± 0.01 ^B,a^	88 ± 4 ^A,b^	99.84 ± 0.01 ^A,a^	81 ± 2 ^A,b^
	WAC (g water⋅g^−1^ protein)		11.5 ± 1 ^A,a^	10.5 ± 0.2 ^A,b^	10.6 ± 1 ^A,a^	8.9 ± 0.2 ^B,b^
	OAC (g oil⋅g^−1^ protein)		1.9 ± 0.2 ^A,a^	1.73 ± 0.02 ^A,b^	1.6 ± 0.1 ^A,a^	1.43 ± 0.03 ^B,b^
	EAI (m^𝟐^ g^−1^)		49 ± 3 ^A,a^	48.1 ± 0.4 ^A,a^	45 ± 4 ^A,a^	17 ± 1 ^B,b^
	ESI (%)		52 ± 3 ^A,a^	48.4 ± 0.2 ^A,b^	44 ± 1 ^B,a^	34 ± 3 ^B,b^
	FAI (%)		43.0 ± 0.1 ^B,a^	43.0 ± 0.1 ^B,a^	71.0 ± 0.1 ^A,a^	68 ± 5 ^A,a^
	FSI (%)	10 min	83.0 ± 0.1 ^A,a^	83.0 ± 0.1 ^A,a^	80.0 ± 0.1 ^B,a^	58 ± 3 ^B,b^
		30 min	83.0 ± 0.1 ^A,a^	67.0 ± 0.1 ^A,b^	50.0 ± 0.1 ^B,a^	42 ± 3 ^B,b^
		45 min	83.0 ± 0.1 ^A,a^	50.0 ± 0.1 ^A,b^	40.0 ± 0.1 ^B,a^	42 ± 3 ^B,a^
		60 min	83.0 ± 0.1 ^A,a^	50.0 ± 0.1 ^A,b^	40.0 ± 0.1 ^B,a^	32 ± 2 ^B,b^

^a^ g kg^−1^ on a dry basis.

^b^ N × 5.46.

^c^ Calculated by mass difference.

^d^ Milligram of gallic acid equivalent per gram of sample.

DSEt, solid defatted with ethanol; DSHx, solid defatted with hexane; TPC, total phenolic compounds; NSI, nitrogen solubility index; WAC, water absorption capacity; OAC, oil absorption capacity; EAI, emulsifying activity index; ESI, emulsifying stability index; FAI, foaming activity index; FSI, foaming stability index; DSEt‐IP/DSHx‐IP, protein obtained by isoelectric precipitation from solid defatted with ethanol or hexane; DSEt‐AP‐N/DSHx‐AP‐N, protein obtained by alcoholic precipitation with neutralized extract from solid defatted with ethanol or hexane. The same lowercase letters within a row do not indicate a significant difference when comparing the same precipitation method across different DSs (*P* > 0.05). The same capital letters in the same row do not differ from each other when comparing different precipitations for the same DS (*P* > 0.05). Comparisons were made between the compositions and between the functionalities of the protein concentrates at the 95% confidence level using a *t*‐test.

The moisture content in the protein concentrates ranged from 120 to 190 g kg^−1^, which is higher than the literature values (27–76 g kg^−1^),^33^, ^34^ even after 96 h of freeze‐drying. The lipid content was less than 12 g kg^−1^, which is less than that reported by Yu *et al*.^34^ (30–170 g kg^−1^). The ash content (14–30 g kg^−1^) decreased relative to that of the DSs (Table 1), and the carbohydrate content ranged from 198–237 g kg^−1^ (Table 3). Wu *et al*.^15^ reported 3.6–179.7 g kg^−1^ carbohydrates in peanut protein concentrates. Alkaline extraction can solubilize non‐protein compounds, whereas ethanol washing can remove sugars and lipids.^1^ However, the low AP temperature (25 °C) likely limited carbohydrate removal, which explains the similarity with IP (*P* > 0.05).

#### Thermal stability of protein concentrates

Table 4 presents the thermal analysis of the protein concentrates, with PPC included for comparison. Because PPC proteins were no treated, their protein fraction likely remained near the native state.

**Table 4:** Differential scanning calorimetry (DSC) results of peanut pressed cake (PPC) and protein concentrates obtained by isoelectric precipitation (IP) and alcoholic precipitation with neutralized extract (AP‐N) from solids defatted with ethanol (DSEt) or hexane (DSHx)

	PPC	Isoelectric precipitation (IP)		Alcoholic precipitation (AP)	
		DSEt‐IP	DSHx‐IP	DSEt‐AP‐N	DSHx‐AP‐N
*T*_d_1 (°C)	95 ± 2 ab	99 ± 2 a	96 ± 1 ab	94 ± 2 b	96 ± 2 ab
Δ*H*_1_ (J g^−1^ protein)	2.4 ± 0.4 a	0.9 ± 0.1 bc	1.6 ± 0.1 ab	0.4 ± 0.1 c	0.8 ± 0.4 bc
*T*_d_2 (°C)	106.6 ± 0.4 c	112 ± 2 a	111 ± 1 ab	110 ± 2 ab	109.4 ± 0.4 b
Δ*H*_2_ (J g^−1^ protein)	17 ± 2 a	4 ± 1 bc	6 ± 1 b	1.6 ± 0.4 c	2 ± 1 c

*T*_d_, temperature of denaturation; Δ*H*, enthalpy of denaturation; PPC, peanut press cake; DSEt‐IP/DSHx‐IP, protein obtained by isoelectric precipitation from solid defatted with ethanol or hexane; DSEt‐AP‐N/DSHx‐AP‐N, protein obtained by alcoholic precipitation with neutralized extract from solid defatted with ethanol or hexane. The same lowercase letters in the same row do not indicate a significant difference (*P* > 0.05). Comparisons were carried out at the 95% confidence level using Duncan's test.

Two thermal events (*T*_d1_ and *T*_d2_) were observed for the PPC and protein concentrates, corresponding to the denaturation of arachin and conarachin, respectively. The literature reports a *T*_d1_ in the range 89.6–93.5 °C for arachin and a *T*_d2_ in the range 101.5–107.3 °C for conarachin.^35^, ^36^ The PPC values (*T*_d1_ = 95 ± 2 °C; *T*_d2_ = 106.6 ± 0.4 °C) align with these ranges. The denaturation temperature reflects protein thermal stability,^37^ and all the protein concentrates presented *T*_d1_ and *T*_d2_ values higher than those in the literature, indicating that the solvents (hexane or ethanol) and precipitation methods (IP and AP) did not compromise stability.

Denaturation enthalpy (Δ*H*) reflects protein structural order.^13^ Compared with PPC, all the concentrates had lower Δ*H* values, indicating that defatting, extraction and purification affect protein structure (Table 4). Among the treatments, DSHx‐IP resulted in the highest ΔH, suggesting that hexane defatting followed by IP is less damaging, which is consistent with the denaturing effect of ethanol during defatting. A comparison of the precipitation methods revealed that compared with IP, AP led to a reduced ΔH, but no significant differences were observed between DSEt‐AP‐N and DSHx‐AP‐N (*P* > 0.05).

#### SDS‐PAGE

The SDS‐PAGE profiles of peanut protein concentrates obtained by IP and AP‐N are shown in Fig. 3, with the subunits classified into five regions: conarachin II (> 50 kDa), arachin acid (39–49.9 kDa), intermediate (23–37.9 kDa), arachin basic (18–22.9 kDa) and conarachin I (14–17.9 kDa).^4^, ^35^, ^36^, ^37^

**Figure 3:**
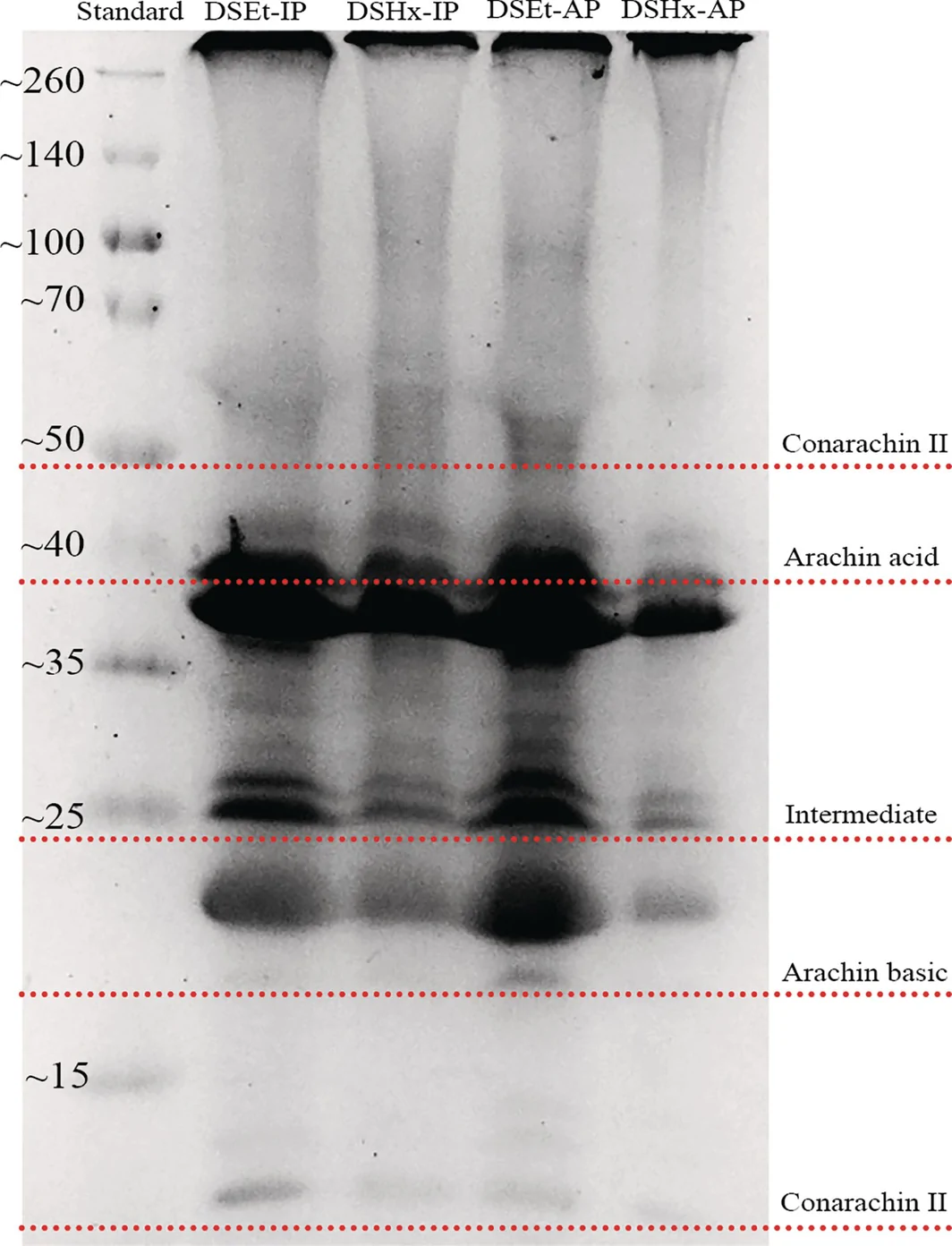
Gel electrophoresis images of sodium‐polyacrylamide dodecyl sulfate from peanut concentrates obtained by isoelectric precipitation (IP) or alcoholic (AP) precipitation from solids defatted with ethanol (DSEt) or hexane (DSHx). The standard particle molar mass was 15–260 kDa.

In the > 50‐kDa region (conarachin II, Ara h1 approximately 61 kDa), DSHx exhibited a lower band intensity than DSEt did, particularly DSHx‐AP‐N, suggesting partial degradation during alcoholic precipitation.

In the 39–50‐kDa region (arachin acid), the DSHx‐IP and DSHx‐AP‐N bands were less intense than those of their DSEt counterparts were, indicating a lower arachin acid content. The use of ethanol may have promoted the aggregation of the more labile hydrolysis subunits, which is consistent with the lower Δ*H*_1_ values observed (Table 4).

The intermediate (approximately 23–38 kDa) and arachin basic (approximately 20 kDa) regions also showed lower intensities for DSHx‐IP and DSHx‐AP‐N, and the DSEt subunits exhibited aggregation in the arachin basic region regardless of the precipitation method.

#### FTIR

FTIR analysis was conducted to evaluate the structural changes in DSs and protein concentrates. The FTIR and second‐derivative spectra of PPC, DSEt, DSHx and the protein concentrates obtained by different purification methods are shown in Fig. 4. The secondary protein structures are observed in the vibrational band corresponding to 1500–1700 cm^−1^ region (amides I and II). Gaussian deconvolution of this region is presented in the Supporting information (Fig. S4). Table 5 summarizes the calculated areas of the protein absorption bands.

**Figure 4:**
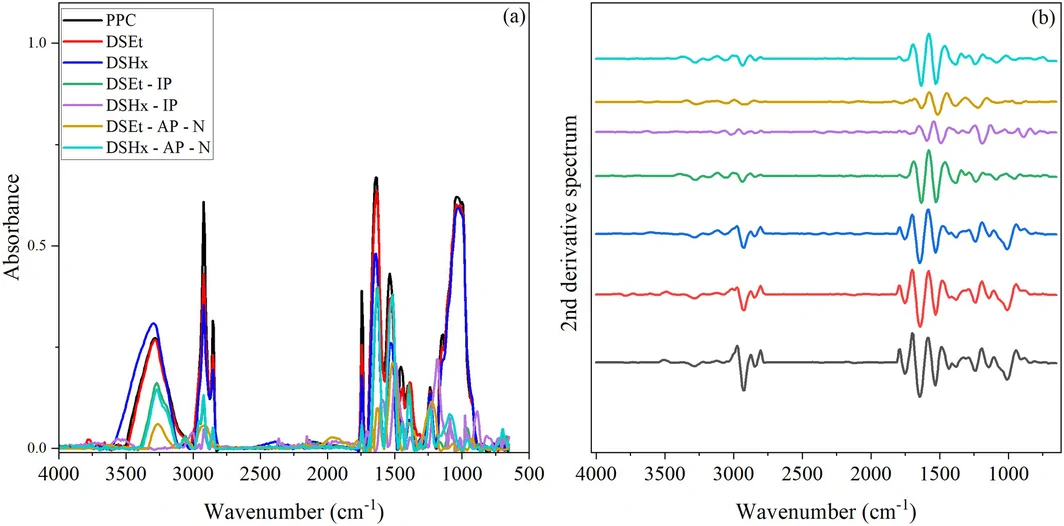
FTIR spectra (a) and second derivative of the spectra (b) for raw material, defatted solids and protein concentrates. PPC, peanut press cake; DSEt, solid defatted with ethanol; DSHx, solid defatted with hexane; DSEt‐IP/DSHx‐IP, protein obtained by isoelectric precipitation from the solid defatted with ethanol or hexane; DSEt‐AP‐N/DSHx‐AP‐N, protein obtained by alcoholic precipitation with the neutralized extract from the solid defatted with ethanol or hexane.

**Table 5:** Absorption regions (amides I and II) resulting from the Gaussian deconvolution of the FTIR spectra of peanut press cake (PPC), ethanol defatted solid (DSEt), hexane defatted solid (DSHx), and protein concentrates obtained by isoelectric (IP) and alcoholic precipitations (AP)

Assignment		PPC		DSEt		DSHx		DSEt‐IP		DSHx‐IP		DSEt‐AP‐N		DSHx‐AP‐N	
Proteins		cm^−1^	Area	cm^−1^	Area	cm^−1^	Area	cm^−1^	Area	cm^−1^	Area	cm^−1^	Area	cm^−1^	Area
Amide II		1514	2.29	1514	2.26	1514	1.97	1513	2.19	‐	‐	1513	1.86	1566	0.80
		1538	10.20	1536	7.54	1537	6.83	1535	7.94	1530	10.37	1532	5.68	1588	6.64
Region area (%)			12.49		9.80		8.80		10.13		10.37		7.54		7.44
Amino acid residue														1609	1.01
Amide I	*β*‐sheets	1630	21.71	1633	27.92	1632	20.73	1625	21.31	1619	12.48	1619	16.15	–	–
	*α*‐helix	1660	13.83	1661	5.59	1661	11.10	1658	16.74	1650	21.83	1652	14.65	–	–
	*β*‐turns	1685	2.03	1682	3.52	1685	2.04	1684	2.46	1682	3.55	1682	2.31	1684	1.00
Region area (%)			37.57		37.03				40.51		37.86		33.11		1.00
Total area (%)			50.06		46.83		42.67		50.64		48.23		40.65		9.45

PPC, peanut press cake; DSEt, solid defatted with ethanol; DSHx, solid defatted with hexane; DSEt‐IP/DSHx‐IP, protein obtained by isoelectric precipitation from solid defatted with ethanol or hexane; DSEt‐AP‐N/DSHx‐AP‐N, protein obtained by alcoholic precipitation with neutralized extract from solid defatted with ethanol or hexane.

The amide II region (1480–1575 cm^−1^) arises from N—H bending and C—N stretching vibrations and is less sensitive to conformational changes.^38^ By contrast, the amide I region (1600–1700 cm^−1^) corresponds to the C=O stretching of peptide bonds and is more sensitive, reflecting alterations in secondary protein structure.^4^, ^39^

Compared with the PPC, the material obtained by defatting with ethanol (DSEt) demonstrated increased *β*‐sheet (from 21.71% to 27.92%) and *β*‐turn (from 2.03% to 3.52%) areas but drastically reduced α‐helix content (from 13.53% to 5.59%), reflecting structural rearrangements because *α*‐helices are highly ordered and stable.^40^ By contrast, DSHx retained a secondary structure similar to that of the PPC. Because *β*‐turns are hydrophilic and *β*‐sheets are hydrophobic,^41^ ethanol induced a new protein conformation that altered the hydrophilic/hydrophobic balance, probably exposing more hydrophilic groups, which is consistent with the moisture and WAC results (Table 1).

The amide II areas (1530 cm^−1^) were markedly lower for DSEt‐AP‐N than for DSEt–IP and DSHx‐IP, whereas no peak was observed for DSHx‐AP‐N (Table 5). Only DSEt‐IP exhibited the *β*‐sheet of amide I (1630 cm^−1^). Because the amide II and *β*‐sheet regions reflect the arachin content,^42^ these findings, along with the results of SDS‐PAGE (Fig. 3), indicate partial degradation of arachin in AP‐treated proteins, particularly DSHx‐AP‐N.

Compared with all the other samples, the AP‐N concentrates had the smallest amide II areas (Fig. 4 and Table 5), and DSHx‐AP‐N had smaller amide I areas, which is consistent with the collapsed protein structure and SDS‐PAGE results. Peak shifts in amide II for DSHx‐AP‐N (1566 and 1588 cm^−1^) suggest structural modifications,^43^ likely resulting from subunit aggregation, whereas a peak at 1609 cm^−1^ may correspond to amino acid residues,^44^ indicating depolymerization or aggregation of the arachin acid subunits, corroborating SDS‐PAGE results.

DSEt‐IP showed a shifts in the *β*‐sheet peaks (1625 cm^−1^), whereas DSHx‐IP and DSEt‐AP‐N displayed shifts in the *β*‐sheet peak (1619 cm^−1^) and *α*‐helix peak (1650–1652 cm^−1^). Considering DSEt, AP decreased the area corresponding to the *β*‐sheet, indicating a decrease in hydrophobicity. With respect to DSHx, AP decreased the areas corresponding to the *β*‐sheet, *β*‐turn and *α*‐helix, indicating a drastic reduction in hydrophobicity and collapsed protein structure.

### Functional properties of the protein concentrates

The functional properties of the peanut protein concentrates (Table 3) were affected by both the extraction method and the precipitation method. The solubility and water and oil absorption capacities are related to the hydration capacity and the exposure of hydrophilic and hydrophobic groups. Proteins from DSEt presented the highest NSI, WAC and OAC values (*P* ≤ 0.05) regardless of the precipitation method, likely because of ethanol‐induced changes in protein conformation during defatting, combined with alkaline pH (9.0) and high temperature (75 °C) during protein extraction.

DSEt‐IP presented the best ESI results, which may be related to the larger area of the *β*‐sheet structure^45^ (Table 5). A comparison of the precipitation methods for DSEt revealed that AP led to lower ESI and FSI than IP did, indicating that the ethanol in the precipitation step reduces hydrophobicity, which is consistent with the FTIR observations.

As discussed in the section on Characterization of protein concentrates, when DSHx‐IP and DSHx‐AP‐N were compared, the use of AP led to a drastic reduction in the amide‐I protein areas (Table 5). Indeed, the WAC, OAC, EAI and ESI values of the DSHx‐AP‐N proteins were less than those of the other protein materials (Table 3), suggesting that the events observed, such as the hydrolysis caused by protein extraction resulting in the subunit aggregation (Fig. 3) associated with the loss and the loss of secondary structures (Table 5), may have negatively affected the functional properties. A comparison of the areas of the α‐helix structure across all the protein materials, revealed that this structure is related to the FSI, as suggested by Sheng *et al*.,^45^ with the DSHx‐AP‐N sample showing the smallest area and the lowest foam stability.

Therefore, the use of ethanol as a precipitating agent may have caused the denaturation of proteins obtained from DSHx because ethanol can solubilize non‐polar side chains, leading to the loss of secondary structures observed in the FTIR results (Table 5).

The WAC and OAC values shown in Table 3 can be compared with those reported by Liu *et al*.,^37^ who obtained peanut protein concentrates through alkaline extraction followed by isoelectric precipitation from pressed cake defatted with hexane, a material equivalent to DSHx‐IP. Liu *et al*.^37^ reported a WAC value of 0.86 g water g^−1^ sample and an OAC value of 3.74 g oil g^−1^ sample. The WAC values reported in Table 3, regardless of the precipitation method, are higher than those reported by Liu *et al*.,^37^ which can be related to the higher carbohydrate contents in protein concentrates (Table 3). Liu *et al*.^37^ reported that different oil extraction and drying methods can affect amino acid composition, surface hydrophilicity and protein conformation.

The FAI values reported in Table 3 were higher than those reported by Liu *et al*.^37^ (22.67). On the other hand, Liu *et al*.^37^ reported higher EAI (86.09 m^2^ g^−1^) and ESI (77.4%) values. Only DSHx‐AP had an FSI value lower than that reported by Liu *et al*.^37^ (70.60%).

Furthermore, the data in Table 3 show that, compared with the use of conventional methods of hexane defatting and isoelectric precipitation (DSHx‐IP), the use of ethanol for both the PPC defatting and protein purification steps (DSEt‐AP‐N) resulted in protein concentrates with greater solubility and FAI values. The WAC, OAC and EAI values of DSEt‐AP‐N are statistically the same as those of DSHx‐IP (*P* > 0.05). This may be related to the structure of the proteins, as indicated by the areas corresponding to *β*‐sheets and *β*‐turns obtained from DSEt ‐AP ‐N (16.15% and 2.31%, respectively) and DSHx‐IP (12.48% and 3.55%, respectively) (Table 5). The *α*‐helix areas for DSHx‐IP (21.83%) and DSEt‐AP‐N (14.65%), and their respective FSI results (Table 3), corroborate the correlation between *α*‐helices and foam stability.

The results indicate that the alternative method for obtaining protein concentrates using ethanol in PPC defatting and protein purification steps (DSEt‐AP‐N) resulted in a product with functional properties similar to those obtained by the traditional procedure (DSHx‐IP), despite the changes observed in protein structure caused by ethanol, but with the advantage of using a renewable and generally recognized as safe (GRAS) solvent.

## CONCLUSIONS

Peanut protein concentrates with high extraction yields were obtained from press cakes defatted with ethanol or hexane at 75 °C under alkaline conditions (pH 9.0). Alcoholic precipitation and isoelectric precipitation yielded concentrates with comparable protein contents. Neutralization of the extract without rotary evaporation was optimal for ethanol‐based precipitation.

Compared with the protein concentrates obtained from hexane defatting and the isoelectric precipitation of proteins (88 ± 4% and 43.0 ± 0.1%, respectively), the protein concentrates obtained using ethanol to defat peanut press cake and to precipitate the protein extracted under alkaline pH showed higher solubility (99.84 ± 0.01%) and FAI (71.0 ± 0.1%) values, reflecting the changes in secondary protein structure observed in the FTIR spectra. FTIR indicated that the use of ethanol during defatting altered the hydrophilic/hydrophobic balance and exposed more hydrophilic groups, regardless of the precipitation method. The ethanol used in the protein purification step (alcoholic precipitation) reduced the protein hydrophobicity, which was more pronounced in hexane‐defatted solids, possibly as a result of the depolymerization or aggregation of the arachin acid subunits.

The extraction conditions also affected the color of the peanut concentrates. Alkaline extraction conditions yielded higher extraction yields but produced redder, darker concentrates than neutral‐pH extractions did. These results suggest that color‐removal strategies, such as adsorption with activated carbon or ion‐exchange resins, could improve the applicability of concentrates obtained under alkaline conditions. On the other hand, protein extraction under neutral pH could be used, despite the lower yields, thereby avoiding the need for pigmentation removal.

Overall, the use of ethanol in both key steps produced protein concentrates with functional properties equivalent to those of conventional hexane‐IP methods, achieving 790 ± 10 g kg^−1^ protein content when a greener, safer solvent was used.

## CONFLICTS OF INTEREST

The authors declare that they have no conflicts of interest.

## DECLARATION OF GENERATIVE AI AND AI‐ASSISTED TECHNOLOGIES IN THE WRITING PROCESS

During the preparation of this work, the authors used Google Translate, Grammarly and ChatGPT to translate and linguistically review the English text. After using this tool/service, the authors reviewed and edited the content as needed and take(s) full responsibility for the content of the publication.

## AUTHOR CONTRIBUTIONS

PJCM, RTP, DG and CEdCR were responsible for conceptualization. PJCM was responsible for data curation. PJCM, LCS, PdCFV, KKA, RTP and MCC were responsible for formal analysis. PJCM. LCS, PdCFV, KKA, RTP and MCC were responsible for investigations. PJCM, KKA, RTP and SMPP were responsible for methodology. PJCM was responsible for software. PJCM, KKA and RTP were responsible for validation. PJCM, MCC and DG were responsible for writing the original draft. CEdCR was responsible for funding acquisition, project administration, resources, supervision and reviewing and editing.

## Supporting information


**Figure S1.** Schematic design of trials highlighting the use of ethanol for oil extraction and for protein precipitation.
**Table S1**. Composition of solid phases (raffinates) obtained from protein extraction from DSEt and DSHx as a function of temperature and pH.
**Figure S2**. Images of protein extracts obtained from DSEt and DSHx as a function of pH and temperature. (a) Protein extraction at 25, 50 and 75 °C at a pH of 6.0; (b) protein extraction at 25, 50 and 75 °C at a pH of 9.0.
**Figure S3**. Photographs of peanut protein concentrates obtained from DSEt and DSHx. (a) Peanut protein concentrates obtained by isoelectric precipitation (IP) and different methods of alcoholic precipitation (AP). (b) Peanut protein concentrates obtained by alcoholic precipitation and neutralized extract (AP‐N) at different agitation times (45, 60, 120 and 180 min).
**Figure S4**. FTIR spectra between 1800 to 1500 cm^−1^ for raw material, defatted solids and protein concentrates (a); (―) PPC; (―) DSEt; (―) DSHx; (―) DSEt‐IP; (―) DSHx‐IP; (―) DSEt‐AP‐N, and (―) DSHx‐AP‐N) and Gaussian deconvolution of PPC (b); DSEt (c); DSHx (d); DSEt‐IP (e); DSHx‐IP (f); DSEt‐AP‐N (g); and DSHx‐AP‐N (h).

## Data Availability

The data that support the findings of this study are available from the corresponding author upon reasonable request.
