# Supplementary material for: Composition and functionalities of peanut protein concentrates using ethanol as a defatted solvent and a precipitating agent
Source: J Sci Food Agric. 2026 Aug 7;106(15):9764–77. doi: 10.1002/jsfa.70967 (PMC13743396; doi:10.1002/jsfa.70967)
Supplement: Supplementary file 1 [file JSFA-106-9764-s001.docx]

**Composition and functionalities of peanut protein concentrates using ethanol as a defatted solvent and a precipitating agent**

Paloma Jamily Cristina MAGALHÃES^a^, Luiza Cardoso STUMM^a^, Paola de Cássia Franco VISIOLI^a^, Keila Kazue ARACAVA^a^, Rafaela Torrezan PRIVATTI^a^, Maria Carolina CAPELLINI^a^, Silvana Marina Piccoli PUGINE^b^, Daniel GONÇALVES^c^, Christianne Elisabete da Costa RODRIGUES^a,^*

***Supplementary Material***

^a^ Laboratório de Engenharia de Separações (LES), Departamento de Engenharia de Alimentos (ZEA), Faculdade de Zootecnia e Engenharia de Alimentos (FZEA), Universidade de São Paulo (USP), P.O. Box 23, 13635-900 Pirassununga, São Paulo, Brazil.

^b^ Laboratório de Química Biológica (LQB), Departamento de Ciências Básicas (ZAB), Faculdade de Zootecnia e Engenharia de Alimentos (FZEA), Universidade de São Paulo (USP), P.O. Box 23, 13635-900 Pirassununga, São Paulo, Brazil.

^c^ Laboratório de Tecnologia de Alimentos (LTA), Centro de Ciências e Tecnologias Agropecuárias (CCTA), Universidade Estadual do Norte Fluminense Darcy Ribeiro (UENF), 28013-602 Campos dos Goytacazes, Rio de Janeiro, Brazil.

*Corresponding author.

chrisrodrigues@usp.br (Christianne E. C. Rodrigues, Ph.D. Professor).

**Figure S1**: Schematic design of trials highlighting the use of ethanol for oil extraction and for protein precipitation.

**Table S1.** Composition of solid phases (raffinates) obtained from protein extraction from DSEt and DSHx as a function of temperature and pH.

| Treatment | | | | Protein  (g⋅kg^-1^)^a,b^ | TPC (mg GAE⋅g^-1^ raffinate)^c^ |
| --- | --- | --- | --- | --- | --- |
| Raw material | pH | | Temperature (°C) |  |  |
| **DSEt** | 6.0 | 25 | | 410 ± 10 ^A,a^ | 1.54 ± 0.03 ^A,b^ |
|  |  | 50 | | 370 ± 20 ^A,a^ | 1.50 ± 0.04 ^A,b^ |
|  |  | 75 | | 420 ± 30 ^A,a^ | 1.25 ± 0.02 ^B,b^ |
|  | 9.0 | 25 | | 200 ± 10 ^A,b^ | 0.34 ± 0.02 ^A,d^ |
|  |  | 50 | | 194 ± 3 ^B,c^ | 0.23 ± 0.01 ^B,d^ |
|  |  | 75 | | 220 ± 10 ^A,b^ | 0.37 ± 0.04 ^A,d^ |
| **DSHx** | 6.0 | 25 | | 410 ± 20 ^A,a^ | 2.6 ± 0.1 ^A,a^ |
|  |  | 50 | | 400 ± 20 ^A,a^ | 1.90 ± 0.04 ^C,a^ |
|  |  | 75 | | 410 ± 20 ^A,a^ | 2.07 ± 0.1 ^B,a^ |
|  | 9.0 | 25 | | 240 ± 20 ^A,b^ | 0.76 ± 0.01 ^B,c^ |
|  |  | 50 | | 260 ± 10 ^A,b^ | 0.82 ± 0.02 ^A,c^ |
|  |  | 75 | | 226 ± 3 ^B,b^ | 0.9 ± 0.1 ^A,c^ |

^a^ g⋅kg^-1^ on dry basis; ^b^ N x 5.46; ^c^ milligram of gallic acid equivalent per gram of raffinate. Same capital letters do not indicate significant difference in the comparison between different temperatures for the same pH. Same lowercase letters do not indicate significant difference in the comparison of the same temperature for different pHs. Comparisons were made at the 95% confidence level by the Duncan’s test.

**Figure S2.** Images of protein extracts obtained from DSEt and DSHx as a function of pH and temperature. (a) Protein extraction at 25, 50, and 75 °C at a pH of 6.0; (b) protein extraction at 25, 50, and 75 °C at a pH of 9.0.

Figure S1 (a): 1 = DSHx at 25 °C; 2 = DSEt at 25 °C; 3 = DSHx at 50 °C; 4 = DSEt at 50 °C; 5 = DSHx at 75 °C; 6 = DSEt at 75 °C.

Figure S1 (b): 7 = DSHx at 25 °C; 8 = DSEt at 25 °C; 9 = DSHx at 50 °C; 10 = DSEt at 50 °C; 11 = DSHx at 75 °C; 12 = DSEt at 75 °C.

**Figure S3.** Photographs of peanut protein concentrates obtained from DSEt and DSHx. (a) Peanut protein concentrates obtained by isoelectric precipitation (IP) and different methods of alcoholic precipitation (AP); (b) peanut protein concentrates obtained by alcoholic precipitation and neutralized extract (AP–N) at different agitation times (45, 60, 120, and 180 minutes).

Figure S2 (a): DSEt = defatted solid with ethanol; DSHx = defatted solid with hexane; DSEt – IP / DSHx – IP, protein obtained from solid defatted with ethanol or hexane by isoelectric precipitation; DSEt – AP / DSHx – AP, protein obtained from solid defatted with ethanol or hexane by alcoholic precipitation; DSEt – AP – N / DSHx – AP – N, protein obtained from solid defatted with ethanol or hexane by alcoholic precipitation with neutralized extract; DSEt – AP – A / DSHx – AP – A, protein obtained from solid defatted with ethanol or hexane by alcoholic precipitation with alkaline extract; DSEt – AP – N – R / DSHx – AP – N – R, protein obtained from solid defatted with ethanol or hexane by alcoholic precipitation with neutralized extract and application of rotaevaporation.

Figure S2 (b): DSEt – AP – N – 45 / DSHx – AP – N – 45, protein obtained from solid defatted with ethanol or hexane by alcoholic precipitation with neutralized extract in 45 minutes of stirring; DSEt – AP – N – 60 / DSHx – AP – N – 60, protein obtained from solid defatted with ethanol or hexane by alcoholic precipitation with neutralized extract in 60 minutes of stirring; DSEt – AP – N – 120 / DSHx – AP – N – 120, protein obtained from solid defatted with ethanol or hexane by alcoholic precipitation with neutralized extract in 120 minutes of stirring; DSEt – AP – N – 180 / DSHx – AP – N – 180, protein obtained from solid defatted with ethanol or hexane by alcoholic precipitation with neutralized extract in 180 minutes of stirring.

**Figure S4.** FTIR spectra between 1800 to 1500 cm^-1^ for raw material, defatted solids and protein concentrates (a; (**―**) PPC; (**―**) DSEt; (**―**) DSHx; (**―**) DSEt – IP; (**―**) DSHx – IP; (**―**) DSEt – AP – N, and (**―**) DSHx – AP – N) and Gaussian deconvolution of PPC (b); DSEt (c); DSHx (d); DSEt – IP (e); DSHx – IP (f); DSEt – AP – N (g), and DSHx – AP – N (h).
